# Estimation of Serum C-terminal Cross-linked Telopeptide Type II Collagen (CTX II) Level to Diagnose Early Knee Osteoarthritis

**DOI:** 10.5704/MOJ.2507.003

**Published:** 2025-07

**Authors:** S Singh, R Khanna, D Jindal

**Affiliations:** 1 Department of Orthopaedics, Teerthanker Mahaveer University Medical College and Research Centre, Moradabad, India; 2 Department of Orthopaedics, Sanjay Gandhi Memorial Hospital, New Delhi, India; 3 Department of Orthopaedics, All India Institute of Medical Sciences, Bilaspur, India

**Keywords:** biomarker, sCTX-II, Cut-off value, osteoarthritis, KOA, KL grade

## Abstract

**Introduction:**

This study aimed to study the usefulness of CTX II levels to identify normal population with patients of knee osteoarthritis, and its utility in identifying the severity of disease in primary knee osteoarthritis (KOA).

**Materials and Methods:**

This research recruited 80 cases of KOA and 80 healthy adults (160 subjects). Patients with primary knee osteoarthritis were graded according to the KL grading system, and serum CTX II (sCTX II) value were analysed. The age, gender, and BMI of the subjects were recorded.

**Results:**

The sCTX-II value in cases (719.87 ± 256.1pg/ml) was more than in controls (419.26 ± 208.18pg/ml, p<0.001). The sCTX-II value in case group was significantly higher in males (812.67 ± 289.24) than in females (680.11 ± 236.59, p=0.03). In the control group, males (426.13 ± 221.06) and females (398.66 ± 166.92) had similar values (p=0.60). sCTX II level was higher with higher age, but this difference is significant in the case group only (p=0.003). Multivariate analysis revealed that the sCTX II level was only dependent on the severity of the disease. Analysis of the ROC curve reveals a cut-off value of sCTX II as 557.5pg/ml among cases and controls, 407.5pg/ml between KL grade 0-I, as 528.5pg/ml between KL grade I-II, as 681.1pg/ml between KL grade II-III, and as 866.4pg/ml between KL grade III-IV.

**Conclusion:**

sCTX II values are dependent only on the severity of the disease. sCTX II level estimation is an excellent diagnostic tool for identifying the normal population with knee osteoarthritis patients and has a clinical significance in identifying KOA cases of KL grade I and II.

## Introduction

Osteoarthritis (OA) is a chronic and progressive disease marked by cartilage and subchondral bone erosion and bony outgrowth at joint margins along with synovial changes. The knee is the most common location of OA due to substantial stresses received by the joint in daily activities and movement^1^. Historically knee osteoarthritis (KOA) was diagnosed clinically by the presence of joint pain, stiffness and limitations of physical function (WOMAC Score)^2^.

In the last five decades, a radiograph of the affected knee was added to grade the degree of involvement or severity of disease as per radiological changes (KL Grading) along with clinical symptoms^3^. Currently, KOA is being diagnosed on a Clinico-radiological basis. However, most patients with KOA are diagnosed late because the patient reports to the clinician only when he becomes symptomatic. Clinical and radiological features are usually not synchronous. Radiological changes always lag behind clinical features^4^. At this point, it is past the stage at which pharmacological treatments will slow or reverse the progression^3^. Hence, in the last couple of decades, scientists have focused on diagnosing KOA much earlier i.e., at molecular or pre-radiological stage of the disease^3,5^. It is at this stage that the persons who are likely to develop the disease soon can be identified and effective interventions of lifestyle changes can be made to avoid the disease^3^. Hence, there is an urgent need for reliable and quantitative which can detect KOA at an early stage^3^. Serological tests can fulfil the aim of diagnosing KOA at the molecular stage and identifying individuals at risk of developing this condition soon^6^.

Destruction of type II collagen is the key event in the pathophysiology of the disease^7^. Among many degradation products of type II collagen, CTX II is abundantly present and is a promising soluble biomarker for diagnosis of OA^6-8^. CTX II is the degradation product of Type II collagen and is the most promising soluble biomarker for diagnosis of KOA^6-8^. Urinary CTX II levels have been extensively studied and have been reported to have very good diagnostic ability in early diagnosis^1,9-11^, prediction of disease in future, OA progression and monitoring health status in population^12^, severity of disease^1,9,13-16^, response to treatment^17-21^ and its ability to enhance accuracy of early diagnosis and a role in clinical utility^22^.

Assessing CTX in a synovial fluid would be ideal as it would be the true representation of the intra-articular environment. Serum and urinary values of CTX II do not truly represent the intra-articular environment as accurately as synovial fluid values^23,24^. Estimation of synovial CTX II value was not considered, as it is an invasive procedure and more difficult to obtain^25^. Hence, the next best logical option is to estimate sCTX in serum. sCTX II estimation kits have been only recently made available for research purpose. Globally, very few studies have been published using this biomarker and none from India. We have tried to document the utility of sCTX II estimation to differentiate between those with and without KOA and also between normal knee (KL grade 0) and that with KL grade I and KL grade II of KOA. This is the first report on the use of serum CTX II as a tool to identify normal knee from KOA knee and the biomarker’s ability to differentiate between disease severity groups from Southeast Asia.

Our aim was to study the usefulness of serum CTX II levels in differentiating between normal and diseased knee and in between all grades of disease severity.

## Materials and Methods

Our study is having level III evidence as it is a case–control study and has approval of the ethical committee and research committee of the institute. It was carried out as per standards laid down Helsinki declaration (1964) and its amendment (2013).

Sample size was determined by a statistical formula (n = Zα/22 ×p× (1-p) / E2), which suggested 80 cases each in control and cases arm. Hence, we included 80 subjects in the cases and control group each, to have a cases and controls in a ratio of 1:1. Eighty adult primary knee osteoarthritis cases who had reported to our orthopaedic out-patient clinic and diagnosed by an orthopaedic consultant. Similarly, 80 adult individuals who did not have any complaints of any joint involvement were recruited as control after verbal and written consent.

Cases of (a) secondary osteoarthritis, (b) primary osteoarthritis of other joints, (c) bleeding disorders, (d) active infection of knee, (e) malignancy, (f) drug abuse, (g) diabetes mellitus, (h) surgical intervention of knee, (i) lactating /lactating mothers, and (j) those not willing to participate were excluded.

All subjects gave a written consent to participate in this study. WOMAC questionnaire was administered to all subjects. Antero-posterior radiograph of knee, in standing position, was taken of all subjects. A senior radiologist, not privy to the clinical features of the subjects, did the KL grading of the radiographs.

About 3ml’s of blood sample were drawn from the medial cubital vein, after overnight fasting and rest for 30 minutes of all subjects. The blood sample was taken in an anticoagulant free test-tubes and incubated for 20 minutes and centrifuged for 10 minutes at 3,000rpm. Supernatant derived was stored at -20° celsius. ELISA technique was used to detect CTX-2 levels in stored serum samples. KinesisDx [Los Angeles, USA] supplied the tests kits.

## Results

Sample size in our study was 160 subjects (80 cases and 80 controls). The subjects in cases group were significantly older than controls. The average age in cases was 52.8±10.31 years (range: 36–78 years) and was 28.55±5.98 years (range: 18–55 years) in the controls (p<0.001). The normal population group has predominance of males (F-20, M-60) while the case group had mostly females (M-24; F-56, p<0.05) (Table I). The BMI (kg/m2) in cases (27.5 ± 4.81) was significantly higher (p<0.05) than with controls (25.4±5.71) (Table II).

**Table I:** sCTX II Levels (pg/ml) among gender groups in subjects.

Gender	sCTX II (pg/ml)								p-value
	Case (n=80)				Controls (n=80)				
	n	Mean	SD	Range	n	Mean	SD	Range	
Male	24	812.67	289.24	296-1403	60	426.13	221.06	142-999.09	p<0.0001
Female	56	680.11	236.59	314-1302	20	398.66	166.92	209.7-738.6	p<0.0001
M v/s F	p= 0.03				p=0.60				

**Table II:** sCTX II Levels (pg/ml) in cases and controls based on BMI.

BMI (kg/m^2^)	sCTX II Levels(pg/ml)						p-value
	Case (N=80)			Controls (N=80)			
	n	Mean	SD	n	Mean	SD	
Underweight (<18.5)	0	0.0	0.0	4	365.55	212.88	-
Normal (18.5-24.9)	27	690.02	278.54	39	427.25	228.41	0.0001
Overweight (25-29.9)	29	759.70	255.6	27	413.20	191.83	0.0001
Obese (≥30)	24	705.34	244.62	10	425.97	192.40	0.003
		p=0.57			p=0.95		

Cases group included 20 subjects of KL grade I, 29 cases in KL grade II, 23 cases in KL grade III, and 8 case in KL grade IV as per disease severity. In the case group, the WOMAC score was 49.27 ± 16.62 and in the control group was zero score. Functional score in KL grade I was recorded as 41.40 ± 16.84 (6.25-70.80), in KL grade II it was 46.78 ± 15.11(18.0-73.9), in KL grade III it was 53.37 ± 14.80 (34.37-83.33) and in KL grade IV it was 66.16 ± 12.76 (43.75-77.00). WOMAC score rises with disease severity (p<0.001).

Analysis of sCTX-II showed that biomarker values in the cases group (range 296-1403; mean: 719.87 ± 256.1pg/ml) were significantly higher (p<0.001) than controls (range: 142-999.09; mean 419.26 ± 208.18pg/ml) (Table III). Biomarker level rises with age in the cases and controls, but the increase was found significant only in the cases (p=0.03) not in normal population group (p=0.737) (Table IV). In the case group the biomarker level (pg/ml) was significantly higher in males (range; 296-1403: mean; 812.67 ± 289.24) than in females (range 314-1302; mean 680.11 ± 236.59) (p=0.03). Similarly, in the controls the biomarker level in males was (range 142-999.09; mean: 426.13 ± 221.06) higher than in females (range: 209.7-738.6; mean: 398.66 ± 166.92) but it was not significant (p=0.60) (Table I). sCTX II level was also analysed for the nutritional status (BMI) of the subjects (cases and controls). It revealed that in various nutritional statuses (underweight, normal, overweight and obese) the biomarker level are generally higher but insignificant in both, the case group (p=0.57) and control group (p=0.95). However, the biomarker value was significantly more (p<0.05) in cases than in controls in each subgroup of BMI i.e. normal, overweight and obese categories (Table II). sCTX II level showed significantly higher values with increasing severity of disease: KL grade 0 (419.26 ± 208.18), KL grade I (533.27 ± 152.19), KL grade II (672.70 ± 159.15), KL grade III (823.46 ± 268.17) and KL grade IV (1059.58 ± 301.3) (Table III).

**Table III:** Serum CTX-2 levels in various KL grades.

	KL grade (n)	sCTX-2 LEVELS (pg/ml) Mean ± SD	t	p-value
1	Controls – Cases (80) – (80)	419.26 ± 208.18 – 719.87 ± 256.1	8.14	p<0.001
2	Grade 0 - Grade I (80) – (20)	419.26 ± 208.18 – 533.27 ± 152.19	2.29	p=0.02
3	Grade I - Grade II (20) – (29)	533.3 ± 152.19 – 672.7 ± 159.15	3.06	p=0.003
4	Grade II - Grade III (29) – (23)	672.7 ± 159.15 – 823.46 ± 268.17	2.52	p=0.01
5	Grade III - Grade IV (23) – (08)	823.5 ± 268.17 – 1059.58 ± 301.3	2.08	P=0.046

**Table IV:** sCTX-2pg/ml in different age groups in cases and controls.

Age (years)	sCTX II Levels(pg/ml)						p-value
	Case (N=80)			Controls (N=80)			
	n	Mean	SD	n	Mean	SD	
18-35	0	0.00	0.00	57	407.75	200.04	306
36-45	8	548.08	203.44	17	444.55	234.18	p=0.3
46-55	20	590.95	144.96	6	456.98	236.05	p=0.09
56-65	28	790.28	280.94	0	0.00	0.00	
> 65	24	802.45	264.3	0	0.00	0.00	
		p=0.003			p=0.737		

Univariate analysis was done to find the associations of CTX II value with WOMAC, age and severity of disease (KL grade). Calculations revealed weak positive correlation (r=0.22) between sCTX-II and WOMAC score, moderate positive correlation (r=0.59) between sCTX-II and KL Grade, a moderate positive correlation (r=0.50) between age and KL Grading, a weak positive co-relation (r=0.219) between age and WOMAC Score and a moderate positive correlation (r=0.50) among sCTX-II and age. Analysis with multiple variables revealed that the CTX II value is dependent on the disease severity only (Table V).

**Table V:** Multivariate analysis of sCTX-2 levels.

	Multivariate Linear SS	Regression DF	Analysis: sCTX-2 (pg/ml) MS	P value
Regression	2116517	7	302360	P<0.0001
Age (years)	6913	1	6913	P=0.6936
Gender	105066	1	105066	P=0.1275
WOMAC Score (%)	10593	1	10593	P=0.6259
KL Grading	1194078	3	398026	P<0.0001
BMI	265.7	1	265.7	P=0.9384

Abbreviations – SS: sum of squares, DF: degrees of freedom, MS: mean of squares

Receiver Operative Curve (ROC) revealed a cut-off value as 557.5pg/ml of sCTX II level between normal population (control) and cases. Similarly, a cut-off value as 407.5pg/ml was shown for KL grades 0 and I, as 528.0pg/ml between KL grades I and II, as 681.1pg/ml between KL grade II and III and as 866.4pg/ml between KL grade III and IV. The sensitivity, specificity, accuracy and discriminating ability as “p-value” are shown in Table VI.

**Table VI:** Cut-off values of sCTX II (pg/ml) between various KL grades.

Between KL grade	Area under curve	Area Under Curve Test Result Variable(s): sCTX II (pg/ml)							
		Standard Error	Asymptotic Significance (p-value)	Asymptotic Lower Bound	95% CI Upper Bound	Cut-off value	Sensitivity	Specificity	Accuracy
Case-Control	0.826	0.032	<0.0001	0.763	0.890	557.5	75.00%	68.75%	70.54%
KL 0-I	0.708	0.053	0.004	0.604	0.812	407.5	90%	57.50%	66.83%
KL I-II	0.743	0.075	0.004	0.594	0.891	528.0	86.21%	60.00%	67.52%
KL II-III	0.667	0.080	0.039	0.509	0.826	681.1	69.57%	62.07%	64.20%
KL III-IV	0.766	0.107	0.027	0.554	0.977	866.4	75.00%	56.52%	61.82%

Notes – A. Under the non-parametric assumption. b. Null hypothesis: true area = 0.5

## Discussion

We did not find any difference in biomarker value between males and females in a normal population. The biomarker value does not vary in a normal population up to 55 years of age (p=0.737), which suggests that probably the biomarker value is independent of the age of the subject. However, we did not have any subject above 55 years of age in the control group and this is one of the limitations of the study (Table IV). We were unable to find any bearing of weight on CTX II values among the normal population (p=0.95). and in the patients (p=0.57). The value of the sCTX II was high (p<0.05) in cases than in controls within each subgroup of BMI i.e. normal, overweight and obese categories (Table II). This suggests that CTX II values are independent of weight. sCTX II shows significantly higher values with increasing severity of disease from KL grade 0 to grade IV. The value of the biomarker is significantly higher in each subsequent group (Table III).

A weak positive correlation of CTX II with WOMAC Score (r=0.22) and moderate positive correlation of CTX II with KL grading (r=0.59) and age (r=0.50) was seen. Positive correlations with age^1,9^, gender^1,9^, BMI1 and disease severity have been reported previously^1,9,14-16,26^. Yet, one researcher has reported a correlation with disease severity but not with age and gender^16^. The cases and control groups were not similar concerning age, gender and BMI. We subjected our data to multivariate analysis and the result suggests that CTX II levels depends only on KL grade not on age, gender and BMI (Table V).

**Fig. 1:**
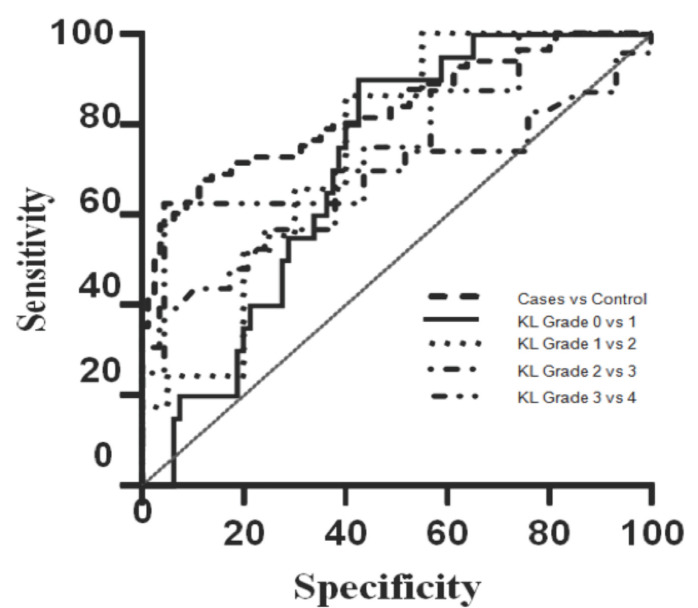
ROC curves of CTX II levels (pg/ml) in various KL grades.

Our extensive search did not reveal any published English language article reporting cut-off points of serum CTX II for various severity grades in primary knee OA. Ours is the first study to document the cut-off levels of sCTX II in healthy population and in various severity grades. ROC curve suggests a cut-off value of 557.5pg/ml to differentiate cases with healthy population with a sensitivity of 75.00%, specificity of 68.75% and accuracy of 70.54%. This value indicates an excellent discriminatory ability (0.0001) of the biomarker between the case and normal population. The ability of the biomarker (CTX II) to differentiate normal population with KOA cases has been documented by many researchers previously as well^1,8,12,24^. The ROC curve was drawn for sCTX II levels among all disease severity grades (KL grade 0 –IV) and their sensitivity and specificity are shown in Fig. 1. Between normal population and grade I cases, the cut-off value is shown as 407.7pg/ml (sensitivity 90%; specificity 57%, p=0.004) and as 528.0pg/ml (sensitivity 86.21%; specificity 60%, p=0.004) in grade I and grade II. This shows that the biomarker has a very good discriminating power. The overlapping in the cut-off value among grade 0 and grade I might be because of the lower number of grade I cases as compared to controls (grade 0).

Further, grade II can be identified by the presence of minimal osteophyte. Similarly, the cut-off value shows robust discriminating ability of the biomarker in grade KL II and III (p=0.039) and in grade KL III and IV (p=0.027). In light of the fact that clinical diagnosis of KOA of KL grade II, III and IV does not pose any difficulty in clinical practice. However, in the absence of clinical signs and symptoms and absence of definite radiological indices both in plain radiography and MRI imaging, identifying the cases of KL grade I is not possible. Hence, with limitations of present-day investigative tools like plain radiography and MRI sCTX II is a cost-effective and a viable diagnostic tool.

There are two limitations in this study, firstly there were no controls above 55 years of age and secondly, the study groups were not matched. Despite these limitations, the results in the present study shows that CTX II estimation is a robust tool to identify cases with controls and could be a promising test to identify those at risk of having the disease in the future. The results of present study should be validated by a study with more precise matching and equal number of the subjects in each group with respect to BMI, gender and age. We suggest that research in future should lay more stress on identifying population at risk (KL grade I) to reduce the burden of disease on the healthcare system.

## Conclusion

Our results showed that the serum CTX II values are dependent only on the severity of disease. It is an excellent tool to differentiate between KOA patients and normal populations. It also shows a robust discriminating power to identify between early KOA cases (KL grade 0, I and II cases).
